# Quantitative analysis of apparent diffusion coefficients to predict neurological prognosis in cardiac arrest survivors: an observational derivation and internal–external validation study

**DOI:** 10.1186/s13054-024-04909-z

**Published:** 2024-04-25

**Authors:** Jung A Yoon, Changshin Kang, Jung Soo Park, Yeonho You, Jin Hong Min, Yong Nam In, Wonjoon Jeong, Hong Jun Ahn, Hye Seon Jeong, Yong Hwan Kim, Byung Kook Lee, Dongha Kim

**Affiliations:** 1 Department of Emergency Medicine, Chungnam National University Hospital, Daejoen, Republic of Korea; 2 Department of Emergency Medicine, College of Medicine, Chungnam National University, 266 Munwha-ro, Jung-gu, Daejeon, 35015 Republic of Korea; 3 Department of Emergency Medicine, Chungnam National University Sejong Hospital, Daejoen, Republic of Korea; 4 Department of Neurology, Chungnam National University Hospital, Daejoen, Republic of Korea; 5 Department of Emergency Medicine, Samsung Changwon Hospital, Sungkyunkwan University School of Medicine, Changwon, Gyeongsangnam-do Republic of Korea; 6 Department of Emergency Medicine, Chonnam National University Medical School, Chonnam National University Hospital, Gwangju, Republic of Korea; 7 Department of Statistics, Sungshin Women’s University, Seoul, Republic of Korea

**Keywords:** Out-of-hospital cardiac arrest, Prognosis, Brain, Magnetic resonance imaging

## Abstract

**Background:**

This study aimed to validate apparent diffusion coefficient (ADC) values and thresholds to predict poor neurological outcomes in out-of-hospital cardiac arrest (OHCA) survivors by quantitatively analysing the ADC values via brain magnetic resonance imaging (MRI).

**Methods:**

This observational study used prospectively collected data from two tertiary academic hospitals. The derivation cohort comprised 70% of the patients randomly selected from one hospital, whereas the internal validation cohort comprised the remaining 30%. The external validation cohort used the data from another hospital, and the MRI data were restricted to scans conducted at 3 T within 72–96 h after an OHCA experience. We analysed the percentage of brain volume below a specific ADC value at 50-step intervals ranging from 200 to 1200 × 10^–6^ mm^2^/s, identifying thresholds that differentiate between good and poor outcomes. Poor neurological outcomes were defined as cerebral performance categories 3–5, 6 months after experiencing an OHCA.

**Results:**

A total of 448 brain MRI scans were evaluated, including a derivation cohort (n = 224) and internal/external validation cohorts (n = 96/128, respectively). The proportion of brain volume with ADC values below 450, 500, 550, 600, and 650 × 10^–6^ mm^2^/s demonstrated good to excellent performance in predicting poor neurological outcomes in the derivation group (area under the curve [AUC] 0.89–0.91), and there were no statistically significant differences in performances among the derivation, internal validation, and external validation groups (all *P* > 0.5). Among these, the proportion of brain volume with an ADC below 600 × 10^–6^ mm^2^/s predicted a poor outcome with a 0% false-positive rate (FPR) and 76% (95% confidence interval [CI] 68–83) sensitivity at a threshold of > 13.2% in the derivation cohort. In both the internal and external validation cohorts, when using the same threshold, a specificity of 100% corresponded to sensitivities of 71% (95% CI 58–81) and 78% (95% CI 66–87), respectively.

**Conclusions:**

In this validation study, by consistently restricting the MRI types and timing during quantitative analysis of ADC values in brain MRI, we observed high reproducibility and sensitivity at a 0% FPR. Prospective multicentre studies are necessary to validate these findings.

**Supplementary Information:**

The online version contains supplementary material available at 10.1186/s13054-024-04909-z.

## Background

For cardiac arrest survivors, the accurate prediction of neurological outcomes serves as an important basis for communicating the patient’s condition to the family and establishing future treatment plans [1–3]. The current international guidelines for post-cardiac arrest care include multiple modalities, including brain imaging techniques such as computed tomography (CT) and magnetic resonance imaging (MRI) [4, 5]. Guidelines suggest that the presence of generalised cerebral oedema, a marked reduction in the grey matter/white matter ratio on brain CT, and extensive diffusion restriction on brain MRI can predict poor neurological outcomes [5–8]. Furthermore, a Korean external validation study reported that a “poor” diffusion-weighted image (DWI) had the highest sensitivity (78%) for predicting poor neurological outcomes, with a 0% false-positive rate (FPR) [9]. Despite the benefits of brain MRI, the qualitative definition in the guidelines lacks objectivity and reproducibility, limiting its use in clinical practice [1, 9–11].

To overcome the limitations associated with the inter-rater reliability of qualitative definitions, a cut-off value has been identified for predicting neurological outcomes through the quantitative analysis of apparent diffusion coefficient (ADC) voxels [12–15]. Among these, in a previous study, Wijman et al. identified the brain volume proportion with an ADC value below 650 × 10^–6^ mm^2^/s with a threshold > 10% as the most efficient parameter for differentiating between good and poor neurological outcomes at 6 months after return of spontaneous circulation (ROSC) [12]. Subsequently, the cut-off value identified in this study was applied to multiple validation studies. However, despite applying the same cut-off values, inconsistent results for area under the curve (AUC; ranging from 0.59 to 0.85), sensitivity (ranging from 59 to 72%), and specificity (ranging from 43 to 96%) values were observed during validation [13–16].

Several hypotheses can explain the inconsistencies in the validation studies. The previously mentioned validation studies employed various methods in MRI analysis. In addition, these studies included data from both 1.5 T and 3 T brain MRIs, with higher magnetic fields generating stronger signals, allowing for higher resolution and faster imaging time [17–19]. Furthermore, a previous study found that hypoxic-ischaemic brain injury (HIBI) progresses over time, causing changes in ADC values on brain MRI, and that DWI imaging for HIBI has the best performance between 2 and 5 days after cardiac arrest [2, 11, 12, 20]. Therefore, the different MRI types and variable imaging timings may have potentially confounded our results. We speculate that these factors hinder the generalisation of the identified cut-off values.

We hypothesised that a reproducible cut-off value could be established by specifying the types of MRI and the timing of image acquisition. We conducted a retrospective analysis of data obtained using a specific type of MRI (3 T) within a specific timeframe (72–96 h after ROSC); both internal and external validation studies were performed using the identified cut-off values. If these values demonstrate high reproducibility, they could serve as a significant predictor in a multimodal approach for prognosis prediction.

## Methods

### Study design and population

This retrospective observational study used a prospectively collected cohort registry from two tertiary academic hospitals (Chungnam National University Hospital [CNUH], Daejeon, Korea, and Samsung Changwon Hospital [SCH], Changwon, Korea). The study period at CNUH was from May 2018 to January 2023, whereas that at SCH was from January 2013 to February 2023. The derivation and internal validation cohorts were randomly composed of 70% and 30% of the patients, respectively, from one hospital (SCH), whereas the external validation cohort was derived from another hospital (CNUH). This study was approved by the institutional review boards of both participating hospitals. Written informed consent was obtained from all patients or their legal guardians before inclusion, and the information was appropriately registered in the database.

Comatose survivors after cardiac arrest were received post-cardiac arrest care (PCAS) bundles, including target temperature management (TTM), except those with active bleeding, refractory hemodynamic instability, possible causes of coma other than cardiac arrest, terminal malignancy, or poor pre-arrest neurological status (Cerebral Performance Category [CPC] 3 or 4), following the current international guidelines [4, 5]. During the study period, TTM was performed at 33 °C or 36 °C, depending on the attending physician. A target temperature of 33 °C or 36 °C was maintained for 24 h using an Arctic Sun® (Energy Transfer Pads™; Medivance Corp, Louisville, CO, USA) feedback-controlled surface cooling device. Upon completion of the TTM maintenance period, the patients were rewarmed to 37 °C at a rate of 0.25 °C/h.

The inclusion criteria were as follows: comatose adults (aged > 18 years) who experienced non-traumatic OHCA, were treated with TTM, and underwent 3 T MRI scan between 72 and 96 h after ROSC. The exclusion criteria were evidence of severe brain atrophy or previous brain injury (ischaemic or haemorrhagic stroke) on MRI, traumatic cardiac arrest, and MRI not performed between 72 and 96 h after ROSC. Additionally, a neurologist, blinded to patient information, reviewed the MRI images to identify patients with serious intracranial metastases and other diseases that could affect the ADC analysis. Consequently, these patients were excluded.

### Data collection

We extracted the following data from the registries of the two participating hospitals: age, sex, comorbidities, cause of cardiac arrest, presence of a witness during collapse, bystander cardiopulmonary resuscitation (CPR), first monitored rhythm, time from collapse to CPR (no-flow time), time from CPR to ROSC (low-flow time), time from ROSC to MRI acquisition, and neurological outcomes at 6 months.

The patient’s neurological status 6 months after ROSC was assessed using their CPC score. This neurological prognostic assessment was conducted through face-to-face visits or standardised follow-up telephone interviews with the patient or a primary caregiver (family member). A poor neurological outcome was defined as a CPC score of 3–5 [21].

Additionally, we retrieved MR images of patients stored in a Picture Archiving and Communication system (PACS) and analysed the ADC values (the method for measuring the ADC values is described below).

### Method for quantitative analysis of ADC value

In both participating hospitals, an MRI scan was performed between 72 and 96 h after ROSC, with the consent of the guardians when the patient’s condition was stable, to assess the extent of HIBI in patients and to share information about the patient’s condition with their family. Therefore, both hospitals had MRI data registries within this specific time range. MRI was performed using a 3 T scanner (Achieva, Philips Healthcare, Amsterdam, Netherlands [CNUH]; Ingenia, Philips Healthcare, Best, Netherlands [SCH]), and included DW-MRI, ADC measurements, and T2-weighted imaging. At CNUH, the MRI protocol included DWI with b-values of 0 and 1000 (TR/TE, 4411.6/46.7 ms; section thickness, 3 mm; section gap, 1 mm; FOV, 240 × 240 mm; matrix, 128 × 126; number of signals acquired, 1) and T2-weighted imaging (TR/TE, 3000/80 ms; section thickness, 5 mm; section gap, 1 mm; FOV, 220 × 220 mm; matrix, 400 × 304; number of signals acquired, 1). Similarly, at SCH, the MRI protocol also included DWI with b-values of 0 and 1000 (TR/TE, 4772.3/85.4 ms; section thickness, 3 mm; section gap, 1 mm; FOV, 240 × 240 mm; matrix, 136 × 131; number of signals acquired, 1) and T2-weighted imaging (TR/TE, 3000/80 ms; section thickness, 5 mm; section gap, 1 mm; FOV, 220 × 220 mm; matrix, 416 × 271; number of signals acquired, 1). These protocols were performed in the axial plane by using 3 orthogonal directions of diffusion-sensitising gradients combined into isotropic images.

For the quantitative analysis of ADC, we employed a recently reported method using automated software (FMRIB Software Library [FSL], Release 5.0 © 2012, The University of Oxford) [2, 15, 16]. ADC MRI images were retrieved in Digital Imaging and Communications in Medicine format from picture archiving and communication system servers at the hospital and were converted to NITFI format using MRIcron (http://www.nitrc.org/projects/mricron). Brain extraction was performed on DWI (b = 1000); eroded brain masks were created using FSL’s Brain Extraction Tool and applied to the ADC maps. Subsequently, the ADC image, obtained after masking, was segmented into three tissue classes: brain parenchyma, CSF components, and remaining extra-soft tissue, using a segmentation technique based on thresholding. To reduce errors caused by artifacts, noise, and fluid contents, voxels with ADC values above 2,000 × 10^–6^ mm^2^/s and below 200 × 10^–6^ mm^2^/s were excluded from the analysis. To establish the threshold range for ADC values, intervals were divided every 50 units within 200 to 1,200 × 10^–6^ mm^2^/s. Subsequently, ADC-R(*x*) was defined by calculating the proportion of the total brain volume occupied by voxels with ADC values ranging from 200 × 10^–6^ mm^2^/s to each threshold. ADC analysis was conducted by an emergency medicine specialist with over 10 years of experience in quantitative MRI analysis using FSL software. The specialist was blinded to the patients’ clinical courses and outcomes.

Ratio of voxels with ADC values ranging from 200 × 10^–6^ mm^2^/s to the threshold (*x*)$ADC - R\left( x \right),\% = \frac{{\sum {\left( {Voxels\;\;~with~\;\;ADC\;\;value\;\;between\;\;~200~\;\;and\;\;x} \right)} }}{{\sum {\left( {Voxels\;\;with~\;\;ADC\;\;value\;\;between\;\;200\;\;~and\;\;2000} \right)} }}~ \times 100$

### Statistical analysis

Counts with percentiles are reported for categorical variables and medians with interquartile ranges (IQRs) for continuous variables because all continuous variables showed a non-normal distribution based on the Shapiro–Wilk test. We compared categorical variables using the χ^2^ tests with continuity correction in 2 × 2 tables or Fisher’s exact test, where appropriate. Continuous variables were compared using the Mann–Whitney *U* test for two groups or the Kruskal–Wallis test with post hoc analysis for three groups. We constructed receiver operating characteristic (ROC) curves to evaluate the prognostic performance of ADC-R(*x*) for neurological outcomes at 6 months. We used ADC-R(x) with the best prognostic performance as the reference and compared other ADC values using the DeLong test with area under the ROC curves (AUC) [22]. Additionally, we conducted predictive performance comparisons of the same ADC-R(x) through the DeLong test in the derivation, internal validation, and external validation cohorts. Subsequently, we set the optimal cut-off value using a specificity of 100% (i.e., an FPR of 0). The AUC, sensitivity, specificity, positive predictive value (PPV), and negative predictive value (NPV) are presented with 95% confidence intervals (CIs). The AUC values of 0.50–0.69, 0.70–0.79, 0.80–0.89, and 0.90–1.00 represent poor, fair, good, and excellent prognostic performance, respectively [23]. We performed statistical analysis using SPSS version 24 (IBM Corp., Armonk, NY, USA) and MedCalc program version 15.2.2 (MedCalc Software, Mariakerke, Belgium), and differences were considered significant at *P* < 0.05.

## Results

### Patient characteristics

**Fig. 1:**
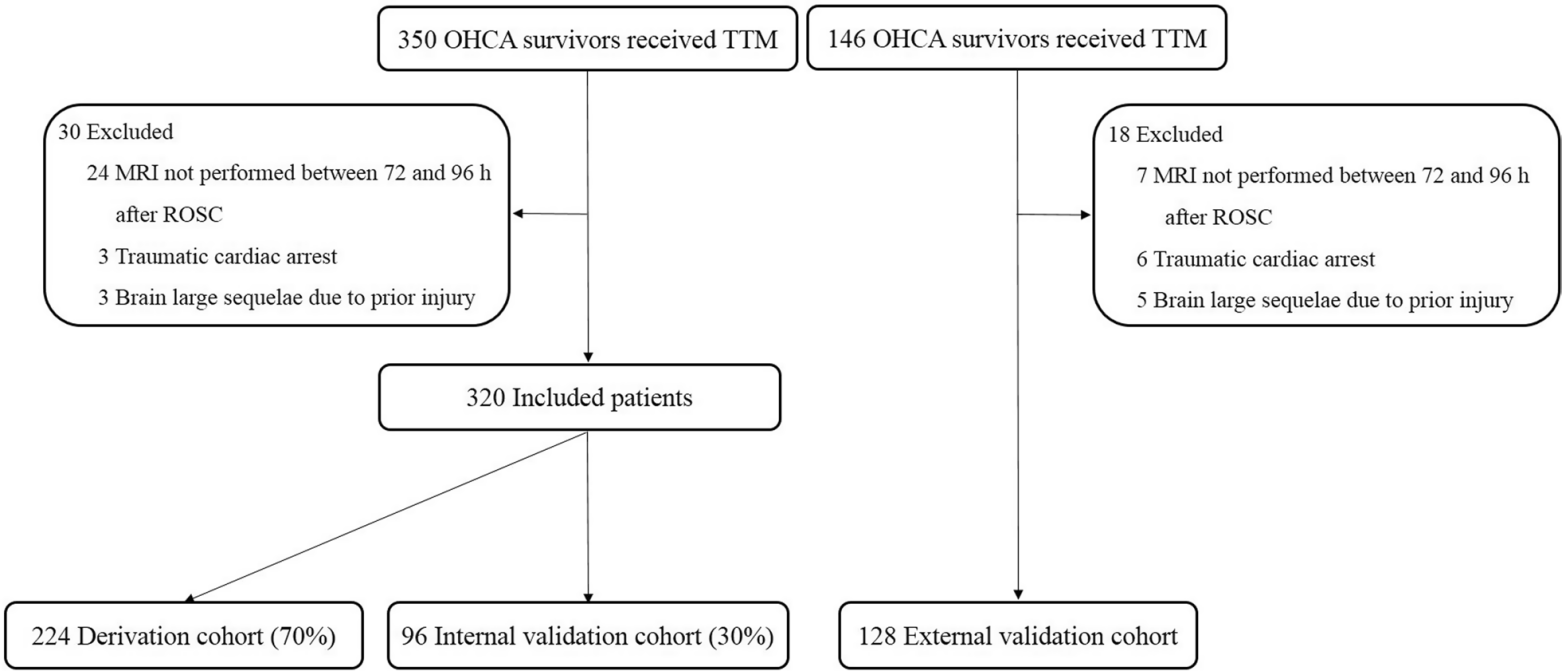
Flow diagram of the patient selection process. OHCA, out-of-hospital cardiac arrest; TTM, target temperature management; MRI, magnetic resonance imaging

During the study period, 496 patients underwent TTM (SCH: n = 350; CNUH: n = 146). Among these patients, 31 did not undergo a brain MRI within 72–96 h from ROSC, 9 had cardiac arrest due to trauma, and 8 showed evidence of prior injury. These 48 patients were excluded, and ultimately, 448 patients were included (SCH, n = 320; CNUH, n = 128) (Fig. 1). The patient demographics and cardiac arrest characteristics are presented in Table 1. The median time from ROSC to MRI scan was 75 h, 76 h and 78 h for the derivation, internal validation, and external validation cohorts, respectively. Compared with the derivation cohort, the internal validation cohort had a higher proportion of individuals with hypertension (*P* = 0.02), while the external validation cohort had a shorter no-flow time (*P* = 0.004) and low-flow time (*P* = 0.009). There were no statistically significant differences in age, sex, presence of other pre-existing illnesses or cardiac arrest characteristics, including witnessed cardiac arrest, bystander CPR, initial shockable rhythm, or rate of cardiac aetiology.

**Table 1:** Baseline demographic data and cardiac arrest characteristics

Characteristics	Derivation cohort, n = 224	Internal validation cohort, n = 96	External validation cohort, n = 128	*P-*value
Age, years	58 (49–67)	58 (45–71)	58 (42–68)	0.90
Female sex	65 (29.0)	28 (29.2)	27 (21.1)	0.18
Preexisting illness				
Coronary artery disease	23 (10.3)	8 (8.3)	20 (15.6)	0.20
Congestive heart disease	7 (3.1)	4 (4.2)	9 (7.0)	0.24
Hypertension	55 (24.6)	38 (39.6)	41 (32.0)	0.02
Diabetes mellitus	47 (21.0)	17 (17.7)	40 (31.3)	0.03
Pulmonary disease	7 (3.1)	6 (6.3)	5 (3.9)	0.41
Renal disease	17 (7.6)	8 (8.3)	19 (14.8)	0.09
Stroke	7 (3.1)	5 (5.2)	6 (4.7)	0.70
Malignancy	14 (6.3)	4 (4.2)	6 (4.7)	0.73
Cardiac arrest characteristics				
Witnessed arrest	126 (56.3)	46 (47.9)	83 (64.8)	0.05
Bystander CPR	132 (58.9)	52 (54.2)	89 (69.5)	0.06
Shockable rhythm	90 (40.2)	31 (32.3)	47 (36.7)	0.39
Cardiac etiology	111 (49.6)	46 (47.9)	56 (43.8)	0.50
No flow time, min	4.0 (1.0–8.0)	6.0 (2.0–10.0)	1.0 (0.0–12.8)^a^	0.004
Low flow time, min	21.0 (11.5–34.5)	25.0 (15.0–42.0)	19.0 (9.0–30.0)^a^	0.009
Time from ROSC to scan, h				
Magnetic resonance imaging	75.0 (73.0–81.0)	76.0 (73.0–81.0)	78.0 (76.0–80.1)^a^	0.001

Continuous and categorical variables are presented as median (interquartile range) and number (%), respectively

^a^*P*-value < 0.017 (= alpha 0.05/3), pairwise multiple comparison with derivation cohort by Kruskal–Wallis test with Mann–Whitney U test after Bonferroni correction

*CPR* Cardiopulmonary resuscitation, *ROSC* Return of spontaneous circulation

### ADC analysis in the derivation cohort

**Fig. 2:**
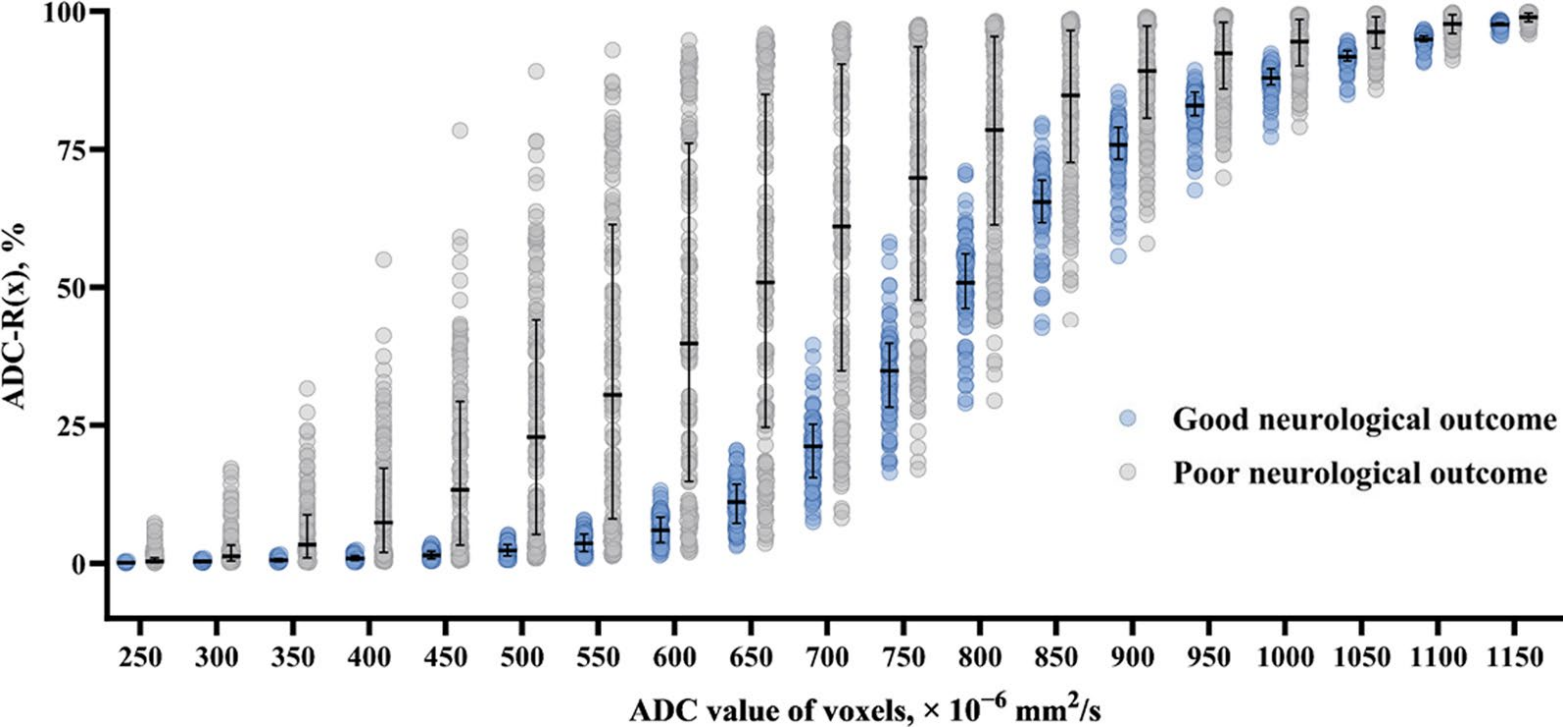
Association between quantitative values of the ADC and neurological outcomes. ADC - R(x) indicates the ratio of voxels with ADC values ranging from 200 × 10^–6^ mm^2^/s to the threshold (x); ADC, apparent diffusion coefficient; error bars, interquartile range; and horizontal lines, median values

In the derivation cohort, the ADC-R(*x*) was significantly higher in the poor neurological outcome group across all ADC value ranges (250–1150 × 10^–6^ mm^2^/s) than that in the good neurological outcome group (all *P* < 0.001; see Fig. 2 and Additional File 1: Table S1). Table 2 highlights the prognostic performance and cut-off values of all ranges of ADC values for poor neurological outcomes 6 months after ROSC. According to the results, ADC-R(500) to ADC-R(650) confers good to excellent prognostic performance. Among the entire range of ADC values, ADC-R(600) exhibited the highest prognostic performance (AUC 0.909; 95% CI 0.863–0.943; cut-off value > 13.2%) and sensitivity (76.1%; 95% CI 68.1–82.9), with a specificity of 100%. When comparing the prognostic performance of ADC-R(600) with other ADC values, there was no statistically significant difference in the prognostic performance between ADC-R(450) and ADC-R(650) (all *P* > 0.05).

**Table 2:** Prognostic performance of specific ADC interval for poor neurological outcome in the derivation cohort

ADC - R (thresholds)^a^	AUC (95% CI)	Cut-off value	Sensitivity (95% CI)	Specificity (95% CI)	PPV (95% CI)	NPV (95% CI)	*P*─value^b^
ADC–R(600)	0.909 (0.863–0.943)	> 13.2	76.1 (68.1–82.9)	100.0 (95.8–100.0)	100	72.3 (65.9–77.8)	Reference
ADC–R(250)	0.813 (0.756–0.862)	> 0.4	45.7 (37.2–54.3)	100.0 (95.8–100.0)	100	53.4 (45.4–61.3)	< 0.001
ADC–R(300)	0.843 (0.789–0.888)	> 0.9	55.1 (46.4–63.5)	100.0 (95.8–100.0)	100	58.1 (49.7–66.2)	0.001
ADC–R(350)	0.870 (0.819–0.911)	> 1.6	65.2 (56.6–73.1)	100.0 (95.8–100.0)	100	64.2 (55.4–72.3)	0.003
ADC–R(400)	0.887 (0.838–0.925)	> 2.4	70.3 (61.9–77.8)	100.0 (95.8–100.0)	100	67.7 (58.8–75.7)	0.04
ADC–R(450)	0.894 (0.846–0.931)	> 3.6	73.9 (65.8–81.0)	100.0 (95.8–100.0)	100	70.5 (61.6–78.4)	0.07
ADC–R(500)	0.901 (0.854–0.937)	> 5.2	75.4 (67.3–82.3)	100.0 (95.8–100.0)	100	71.7 (62.7–79.5)	0.16
ADC–R(550)	0.907 (0.861–0.942)	> 7.9	76.1 (68.1–82.9)	100.0 (95.8–100.0)	100	72.3 (65.9–77.8)	0.56
ADC–R(650)	0.902 (0.855–0.937)	> 20.5	76.8 (68.9–83.6)	100.0 (95.8–100.0)	100	72.9 (66.5–78.5)	0.09
ADC–R(700)	0.890 (0.841–0.928)	> 39.6	69.6 (61.2–77.1)	100.0 (95.8–100.0)	100	67.2 (58.3–75.2)	0.02
ADC–R(750)	0.873 (0.822–0.913)	> 58.2	64.5 (55.9–72.4)	100.0 (95.8–100.0)	100	63.7 (55.0–71.8)	0.007
ADC–R(800)	0.857 (0.804–0.900)	> 71.1	63.8 (55.2–71.8)	100.0 (95.8–100.0)	100	63.2 (54.5–71.3)	0.002
ADC–R(850)	0.848 (0.794–0.892)	> 79.7	61.6 (52.9–69.7)	100.0 (95.8–100.0)	100	61.9 (53.3–70.0)	0.002
ADC–R(900)	0.842 (0.788–0.887)	> 85.4	60.9 (52.2–69.1)	100.0 (95.8–100.0)	100	61.4 (52.8–69.5)	0.002
ADC–R(950)	0.843 (0.788–0.888)	> 89.4	63.8 (55.2–71.8)	100.0 (95.8–100.0)	100	63.2 (54.5–71.3)	0.003
ADC–R(1000)	0.842 (0.787–0.887)	> 92.3	63.0 (54.4–71.1)	100.0 (95.8–100.0)	100	62.8 (54.1–70.9)	0.004
ADC–R(1050)	0.841 (0.787–0.887)	> 94.7	63.0 (54.4–71.1)	100.0 (95.8–100.0)	100	62.8 (54.1–70.9)	0.004
ADC–R(1100)	0.842 (0.788–0.887)	> 96.8	63.0 (54.4–71.1)	100.0 (95.8–100.0)	100	62.8 (54.1–70.9)	0.005
ADC–R(1150)	0.842 (0.787–0.887)	> 98.5	62.3 (53.7–70.4)	100.0 (95.8–100.0)	100	62.3 (53.7–70.4)	0.006

^a^The definition of ADC - R(*x*) is the ratio of voxels with ADC values ranging from 200 × 10^−6^ mm^2^/s to the threshold (*x*)

^b^*P* values are based on the DeLong test for comparison of the area under the receiver operating characteristic curve (reference: ADC–R[600])

*ADC* Apparent diffusion coefficient, *AUC* Area under the receiver operating characteristic curve, *CI* Confidence interval, *PPV* Positive predictive value, *NPV* Negative predictive value

### ADC analysis in internal and external validation cohorts

**Fig. 3:**
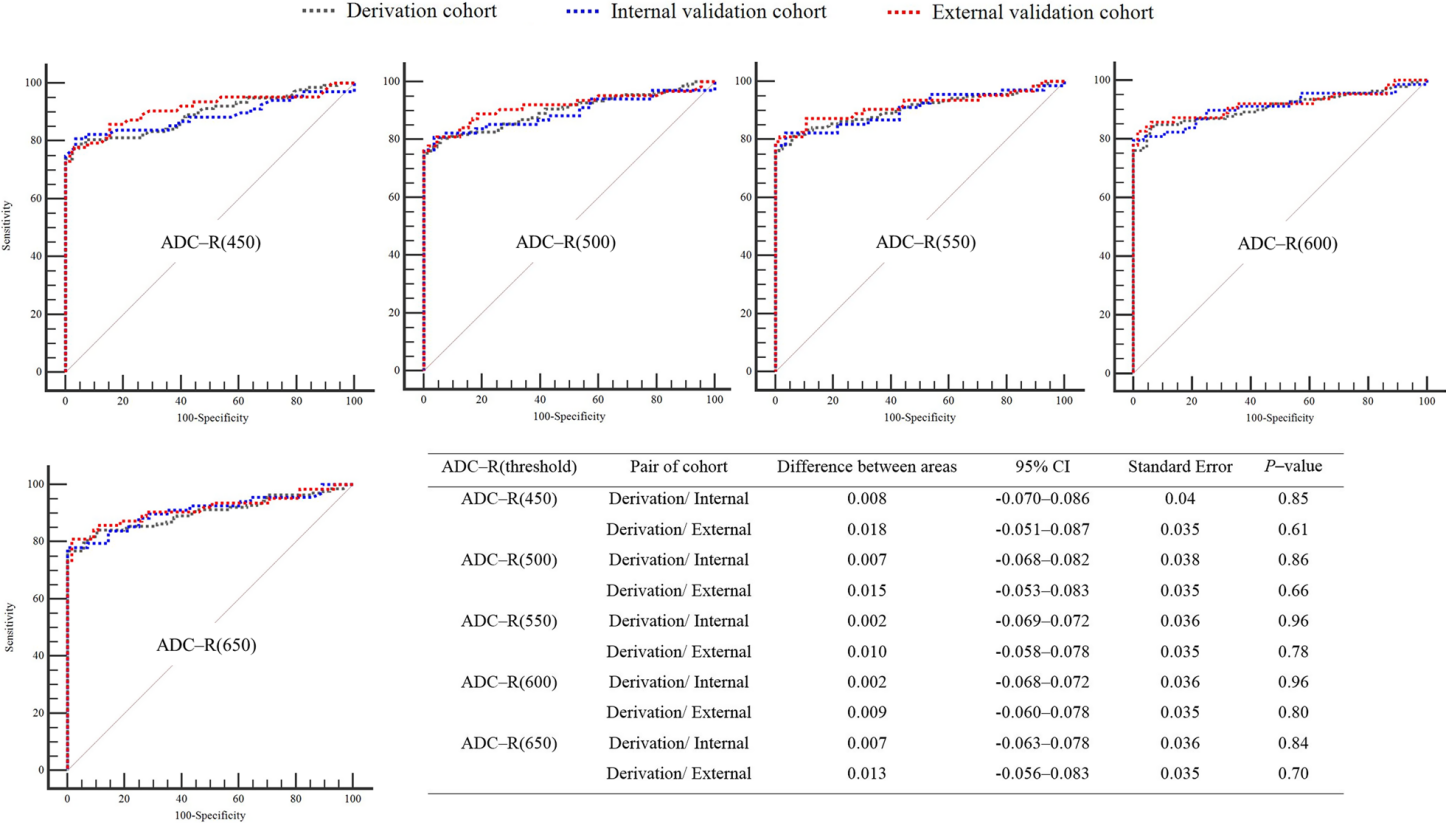
Pairwise comparison of ROC curves between derivation and internal/external validation cohorts for ADC-R(450) to ADC-R(650). ADC-R(x) indicates the ratio of voxels with ADC values ranging from 200 × 10^–6^ mm^2^/s to the threshold (*x*); ADC, apparent diffusion coefficient; CI, confidence interval; ROC, receiver operating characteristic

The prognostic performance of ADC-R(450) to ADC-R(650) 72–96 h after ROSC for a poor neurological outcome at 6 months showed no significant differences between the internal and external validation cohorts compared to that in the derivation cohort (all *P* > 0.6; Fig. 3). Among them, ADC-R(600) showed the best-similar prognostic performance (*P* ≧ 0.8). Thus, the cut-off values obtained from ADC-R(450) to ADC-R(650) in the derivation cohort were applied to the internal and external cohorts (Table 3). Between ADC-R(500) to ADC-R(650), the internal validation cohort exhibited a sensitivity of 70.6% (95% CI 58.3–81.0) with 100% specificity. In the external validation cohort, intervals excluding ADC-R(650), showed high sensitivity and 100% specificity, similar to the derivation cohort. Among them, ADC - R(600) demonstrated the highest values with a sensitivity of 77.8% (95% CI 65.5–87.3) with 100% specificity, when using a cut-off value of > 13.2%. When the previously suggested ADC-R(650) cut-off of > 10% was applied, it predicted a poor neurological outcome with a specificity of 39.5% (95% CI 29.2–50.7) and a sensitivity of 92.0% (95% CI 86.2–96.0) in the derivation cohort, a specificity of 46.4% (95% CI 27.5–66.1) and a sensitivity of 92.7% (95% CI 83.7–97.6) in the internal validation cohort, and a specificity of 33.9% (95% CI 22.6–46.6) and a sensitivity of 93.7% (95% CI 84.5–98.2) in the external validation cohort (Table 4).

**Table 3:** Application of cutoff values from ADC - R(450) to ADC - R(650) in the derivation cohort to the internal and external Cohorts

ADC - R(thresholds)^a^	Cut-off value	AUC (95% CI)	Sensitivity (95% CI)	Specificity (95% CI)	PPV (95% CI)	NPV (95% CI)
*Derivation cohort*						
ADC–R(450)	> 3.6	0.894 (0.846–0.931)	73.9 (65.8–81.0)	100.0 (95.8–100.0)	100	70.5 (64.3–76.0)
ADC–R(500)	> 5.2	0.901 (0.854–0.937)	75.4 (67.3–82.3)	100.0 (95.8–100.0)	100	71.7 (65.4–77.2)
ADC–R(550)	> 7.9	0.907 (0.861–0.942)	76.1 (68.1–82.9)	100.0 (95.8–100.0)	100	72.3 (65.9–77.8)
ADC–R(600)	> 13.2	0.909 (0.863–0.943)	76.1 (68.1–82.9)	100.0 (95.8–100.0)	100	72.3 (65.9–77.8)
ADC–R(650)	> 20.5	0.902 (0.855–0.937)	76.8 (68.9–83.6)	100.0 (95.8–100.0)	100	72.9 (66.5–78.5)
*Internal validation cohort*						
ADC–R(450)	> 3.6	0.886 (0.805–0.942)	66.2 (53.7–77.2)	100.0 (87.7–100.0)	100	54.9 (46.6–62.9)
ADC–R(500)	> 5.2	0.894 (0.815–0.948)	70.6 (58.3–81.0)	100.0 (87.7–100.0)	100	58.3 (49.2–66.9)
ADC–R(550)	> 7.9	0.905 (0.829–0.956)	70.6 (58.3–81.0)	100.0 (87.7–100.0)	100	58.3 (49.2–66.9)
ADC–R(600)	> 13.2	0.911 (0.835–0.959)	70.6 (58.3–81.0)	100.0 (87.7–100.0)	100	58.3 (49.2–66.9)
ADC–R(650)	> 20.5	0.909 (0.833–0.958)	70.6 (58.3–81.0)	100.0 (87.7–100.0)	100	58.3 (49.2–66.9)
*External validation cohort*						
ADC–R(450)	> 3.6	0.912 (0.849–0.955)	66.7 (53.7–78.0)	100.0 (94.5–100.0)	100	75.6 (68.6–81.4)
ADC–R(500)	> 5.2	0.916 (0.854–0.958)	73.4 (60.3–83.4)	100.0 (94.5–100.0)	100	79.3 (71.8–85.2)
ADC–R(550)	> 7.9	0.917 (0.855–0.958)	74.6 (62.1–84.7)	100.0 (94.5–100.0)	100	80.2 (72.7–86.1)
ADC–R(600)	> 13.2	0.918 (0.856–0.959)	77.8 (65.5–87.3)	100.0 (94.5–100.0)	100	82.3 (74.5–88.1)
ADC–R(650)	> 20.5	0.915 (0.853–0.957)	79.4 (67.3–88.5)	98.5 (91.7–100.0)	98.0 (87.7–99.7)	83.1 (75.2–88.9)

^a^The definition of ADC - R(*x*) is the ratio of voxels with ADC values ranging from 200 × 10^−6^ mm^2^/s to the threshold (*x*)

*ADC* Apparent diffusion coefficient, *AUC* Area under the receiver operating characteristic curve, *CI* Confidence interval, *PPV* Positive predictive value, *NPV* Negative predictive value

**Table 4:** Comparison of prognostic performance of ADC - R(650) cutoff of > 10% and > 20.5% in derivation, internal validation, and external validation cohorts

Cohorts	Cut-off value, ADC - R(650)^a^	FP	FN	TP	TN	Sensitivity (95% CI)	Specificity (95% CI)	PPV (95% CI)	NPV (95% CI)
Derivation	> 10%	53	11	127	33	92.0 (86.2─96.0)	39.5 (29.2─50.7)	70.9 (67.2─74.5)	75.6 (62.3─85.2)
	> 20.5%	0	33	107	85	76.8 (68.9─83.6)	100 (95.8─100.0)	100	72.9 (66.5─78.5)
Internal validation	> 10%	16	5	63	12	92.7 (83.7─97.6)	46.4 (27.5─66.1)	80.8 (74.7─85.6)	72.2 (50.6─86.9)
	> 20.5%	0	19	49	28	66.2 (53.4─77.4)	100 (88.8─100.0)	100	58.5 (50.1─66.4)
External validation	> 10%	44	4	59	21	93.7 (84.5─98.2)	33.9 (22.6─46.6)	57.8 (53.5─62.3)	84.6 (66.8─93.8)
	> 20.5%	1	12	51	64	79.4 (67.3─88.5)	98.5 (91.7─100.0)	98.0 (97.7─99.7)	83.1 (75.2─88.9)

^a^The definition of ADC - R(650) is the ratio of voxels with ADC values ranging from 200 × 10^−6^ mm^2^/s to the 650 × 10^−6^ mm^2^/s

*FP* number of patients with a false-positive test result, *FN* Number of patients with a false-negative test result, *TP* Number of patients with a true positive test result, *TN* number of patients with a true negative test result, *CI* Confidence interval, *PPV* Positive predictive value, *NPV* Negative predictive value

## Discussion

In this retrospective multicentre registry-based cohort study, we found that MRI demonstrated a high prognostic performance for poor neurological outcomes, exhibiting a sensitivity of over 70% when the FPR was 0%. We also observed that maintaining consistent specifications for the MRI type (3 T) and the timing of image acquisition (72–96 h after ROSC) resulted in high reproducibility. Notably, the ADC-R(600) showed the highest reproducibility and sensitivity (77.8% when FPR was 0%) in the external validation cohort. Furthermore, applying the previously suggested cut-off value of > 10% to our validation cohort, ADC-R(650) resulted in 49% sensitivity when the FPR was 3%, suggesting the need to propose a new cut-off value. This consideration should include the type (1.5 T vs. 3 T) and timing of MRI acquisition.

International guidelines for post-cardiac arrest care recommend a multimodal neuroprognostic strategy at 72 h after ROSC, rather than using a single factor to predict neurological outcomes which may not be 100% accurate and lead to false positives [5, 6]. However, obtaining all the desired predictors is not always possible when predicting prognosis, and the best combination to increase predictive performance is not known [9, 24–26]. Recently, an observational study prospectively collected data from 130 patients with OHCA and conducted external validation of the 2020 European Resuscitation Council and the European Society of Intensive Care Medicine prognosis algorithm to predict neurological outcomes using a combination strategy [26]. This study showed that indiscriminately adding predictive variables did not enhance the prognostic performance or efficiency. However, when the MRI results were considered, the sensitivity significantly improved in predicting poor neurological outcomes when the FPR was 0%. When applied clinically, MRI can be performed in a blinded state and is unaffected by sedatives or neuromuscular blockers administered to the patients. However, the lack of measurement standards and limited number of studies have hindered the reproducibility of the results [5, 27–31]. Moreover, this approach may not be feasible for unstable patients. International guidelines recommend the use of MRI for prognosis only in centres with a specific expertise [5].

Efforts have been made to quantitatively analyse the percentage of brain volume below each voxel value in ADC MR images to predict neurological outcomes [2, 12–15]. The goal was to overcome the limitations of qualitative analysis (presence or absence of high signal intensity), including ambiguity and difficulty with inter-rater reliability [10, 31–35]. However, a clear cut-off value has not yet been proposed. The most commonly used cut-off value is based on the results of a prospective single-centre study involving 51 patients [12], which showed that the optimal cut-off value for predicting neurological outcomes 6 months after cardiac arrest is when the proportion of brain volume with an ADC below 650 × 10^–6^ mm^2^/s exceeds 10%. This demonstrated a predictive value for death or vegetative state with a specificity of 100% and a sensitivity of 81%. Subsequently, several validation studies were conducted to determine cut-off values [13–15]. In the validation study conducted by Hirsch et al., which involved 51 patients, the predictive value ranged from poor to excellent, with an AUC of 0.79 (95% CI 0.65–0.93) [15]. When the presented cut-off value was applied, it showed a sensitivity of 63% (95% CI 0.42–0.80) and a specificity of 96% (95% CI 0.77–0.99). However, in another multicentre study by Hirsch et al. involving 125 patients, despite applying the same cut-off value, the AUC was 0.85 (0.78–0.91), with a sensitivity of 72% (95% CI 61–80), and a specificity of 91% (95% CI 75–98) [14]. Furthermore, in a separate multicentre study by Wouters et al., the same cut-off value was applied to 58 patients, resulting in an AUC of 0.59 (95% CI 0.45–0.72), a sensitivity of 59%, and a specificity of 43% [13]. This demonstrated a lower predictive power compared to that of other validation studies. In our cohort, when the same cut-off value was applied, the sensitivity and specificity for predicting poor outcomes 6 months after ROSC were 39.5% (95% CI 29.2–50.7) and 92.0% (95% CI 86.2–96.0) in the derivation group, and 46.4% (95% CI 27.5–66.1) and 92.7% (95% CI 83.7–97.6) in the external validation group, respectively. This predictive performance was significantly lower than the sensitivity of 76.8% and specificity of 100% in the derivation group when the ADC value exceeded the cut-off value of 20.5% at ADC-R(650) in our study.

Despite applying the initially proposed criteria identically, the consistency of the results across the studies was low [13–15]. However, our study demonstrated high reproducibility with a high degree of agreement in the derivation, internal validation, and external validation cohorts. Therefore, we hypothesise the following: First, in the aforementioned studies, the quantitative analysis of ADC values was conducted using various tools and methods, leading to inconsistent results. Differences in the MRI analysis software can affect the quantification of ADC values in voxels, and the absence of standardised analysis methods may pose limitations in deriving an optimal cut-off value [12–15]. Therefore, in our study, we conducted a voxel-based analysis using the FSL software, as demonstrated by Moon et al., to predict the neurological outcome of cardiac arrest survivors [16]. Second, the results obtained using different types of MRI may compromise the accuracy of the optimal cut-off value. According to previous studies on MRI, 3 T MRI has twice the signal-to-noise ratio of 1.5 T MRI. A higher signal-to-noise ratio either reduces the scan time or obtains high-resolution images, thereby increasing the temporal and spatial resolutions of the images [17–19]. Wijman et al., who proposed the cut-off value of 10% for the proportion of brain volume with an ADC below 650 × 10^–6^ mm^2^/s, utilised only a 1.5 T scanner [12]. However, in the subsequent validation studies, both 1.5 T and 3 T scanners were used [13–15]. Assuming this could impact the results, we exclusively utilised images obtained from a single type of 3 T MRI. Third, according to one of our institution’s previous studies, which quantitatively analysed ADC images from the first MRI performed within 6 h of ROSC and the second MRI performed within 72 and 96 h of ROSC, HIBI progresses over time, and this change is reflected in the ADC images [2]. Therefore, we confirmed a statistically significant increase in the proportion of voxels with ADC values up to each threshold, across the entire brain. This indicates that the quantitative values for HIBI obtained by analysing ADC images are time-dependent. The wide distribution of MRI acquisition times within seven days of cardiac arrest in existing validation studies thus could have influenced the sensitivity and specificity of the results. Therefore, we only included images obtained within 72 and 96 h of ROSC in our study.

Our study has two strengths compared to previous studies. First, this study included 448 patients with OHCA who underwent brain MRI. When performing MRI on patients who have recovered from cardiac arrest, there are various limitations, such as patient stability, difficulty in moving during the examination, and cost. Considering the constraints of scanner type and time, this study, which included more than 400 patients, cannot be considered to have a small sample size. Second, in this cohort, the number of patients whose MRI performance time fell outside the 72–96 h window after ROSC during the study period was small (31 patients, 6%), which helped reduce the error of selection bias. Despite these strengths, our study has several limitations. First, although it was a retrospective, multicentre, registry-based cohort study, all participants were limited to being from two tertiary university hospitals in Korea, which may limit the generalizability of the study results. This raises questions about the applicability of the results to other ethnic groups, and additional prospective multicentre studies are needed to validate the results across diverse demographic groups. Second, during the study period, of the 558 patients who had an indication for TTM after achieving ROSC, 62 (12.0%) were excluded because they did not undergo TTM. This could have caused a selection bias, which may limit the generalizability of the study results. Third, it is difficult to perform MRI scans in critically ill patients; although this study used an analysis method proven to predict neurological outcomes, there is currently no universal consensus on MRI analysis. Therefore, there are limitations to applying the results of this study to general clinical practice. Fourth, in this study, the two hospitals used MRI scanners of different models but from the same vendor (Philips Healthcare, Netherlands). Indeed, variations in ADC measurements can occur due to different MRI vendors, but the inter-scanner coefficients of variation for overall gray matter and white matter on ADC and mean diffusivity are relatively low (< 4%) [36, 37]. To generalise the findings of this study, further comparative research is needed, where groups using scanners from different vendors are compared under the same settings (3 T MRI, MRI scans performed between 72 and 96 h after ROSC). Fifth, this study was conducted in a population where the causes of cardiac arrest were not solely cardiac-related but also included respiratory or mixed aetiologies. This may increase the dispersion of the results. Despite these limitations, the results of an MRI scan can be quantified when predicting the prognosis of cardiac arrest survivors, showing a high sensitivity when the FPR is 0%. Using a multimodal approach enhances the predictive performance when combined with other predictors. As such, further research is required for appropriate generalisation.

## Conclusions

In conclusion, the quantitative analysis values obtained using ADC from a 3 T MRI scanner performed between 72 and 96 h after ROSC demonstrated high sensitivity, excellent predictive performance, and high reproducibility when predicting poor neurological outcomes six months later, especially when the FPR was 0%. In particular, the proportion of brain volume with an ADC range below 450 to 650 × 10^–6^ mm^2^/s showed the best predictive performance and reproducibility. Furthermore, the previously proposed suggestion that poor neurological outcomes are likely when exceeding 10% of the proportion of brain volume with an ADC below 650 × 10^–6^ mm^2^/s implies that a new cut-off value may be necessary to improve predictive performance. Additional validation studies are needed to evaluate whether the cut-off values obtained using these specific MRI types and acquisition time points can enhance the performance of prognostic strategy algorithms after cardiac arrest.

### Supplementary Information

**Additional file 1. Table S1.** Associations between the voxel-based quantitatively analyzed parameters of ADC and neurological outcomes in the derivation cohort.

## Data Availability

The datasets used and/or analysed during the current study are available from the corresponding author on reasonable request.

## Abbreviations

ADC: Apparent diffusion coefficient
AUC: Area under the curve
CPC: Cerebral performance category
CPR: Cardiopulmonary resuscitation
CT: Computed tomography
DWI: Diffusion-weighted image
FPR: False-positive rate
HIBI: Hypoxic-ischaemic brain injury
IQR: Interquartile range
MRI: Magnetic resonance imaging
NPV: Negative predictive value
PCAS: Post-cardiac arrest care
PPV: Positive predictive value
ROSC: Return of spontaneous circulation
