# Supplementary material for: Quantitative analysis of apparent diffusion coefficients to predict neurological prognosis in cardiac arrest survivors: an observational derivation and internal–external validation study
Source: Crit Care. 2024 Apr 25;28:138. doi: 10.1186/s13054-024-04909-z (PMC11044301; doi:10.1186/s13054-024-04909-z)
Supplement: Supplementary file 1 — Additional file 1. Table S1. Associations between the voxel-based quantitatively analyzed parameters of ADC and neurological outcomes in the derivation cohort. [file 13054_2024_4909_MOESM1_ESM.docx]

**Supplemental materials**

**Title:** **Quantitative Analysis of Apparent Diffusion Coefficients to Predict Neurological Prognosis in Cardiac Arrest Survivors: An Observational Derivation and Internal-External Validation Study**

**eAppendix 1. Table S1.** Associations between the voxel-based quantitatively analyzed parameters of ADC and neurological outcomes in the derivation cohort

**1. Table S1.** **Associations between the voxel-based quantitatively analyzed parameters of ADC and neurological outcomes in the derivation cohort**

| ADC−R(thresholds)^a^ | | Overall cohort, n = 224 | Good neurological outcome, n = 83 | Poor neurological outcome, n = 141 | *P*-value^b^ |
| --- | --- | --- | --- | --- | --- |
| Thresholds  of ADC,  × 10^−6^ mm²/s | ADC−R(250) | 0.21 (0.11–0.59) | 0.13 (0.08–0.20) | 0.35 (0.16–0.99) | <0.001 |
|  | ADC−R(300) | 0.53 (0.28–2.06) | 0.32 (0.18–0.48) | 1.10 (0.45–3.30) | <0.001 |
|  | ADC−R(350) | 1.00 (0.52–5.32) | 0.56 (0.30–0.84) | 2.95 (0.95–8.59) | <0.001 |
|  | ADC−R(400) | 1.80 (0.87–10.74) | 0.88 (0.49–1.32) | 6.44 (1.80–17.50) | <0.001 |
|  | ADC−R(450) | 2.98 (1.42–20.82) | 1.42 (0.82–2.05) | 12.03 (3.10–29.23) | <0.001 |
|  | ADC−R(500) | 4.42 (2.31–29.34) | 2.25 (1.34–3.18) | 20.20 (4.82–42.14) | <0.001 |
|  | ADC−R(550) | 7.00 (3.59–39.23) | 3.54 (2.19–5.07) | 29.32 (7.38–57.21) | <0.001 |
|  | ADC−R(600) | 10.66 (6.05–50.35) | 5.93 (3.87–8.04) | 38.20 (11.45–68.68) | <0.001 |
|  | ADC−R(650) | 18.26 (11.11–60.87) | 10.72 (7.31–13.91) | 49.39 (18.85–77.65) | <0.001 |
|  | ADC−R(700) | 29.89 (20.34–68.53) | 20.32 (15.37–23.82) | 59.05 (32.26–84.80) | <0.001 |
|  | ADC−R(750) | 44.08 (33.91–75.18) | 34.22 (27.94–39.19) | 68.93 (45.11–89.66) | <0.001 |
|  | ADC−R(800) | 59.37 (50.35–81.42) | 50.68 (45.54–55.47) | 77.11 (58.86–92.81) | <0.001 |
|  | ADC−R(850) | 71.32 (64.25–87.12) | 64.81 (61.66–69.04) | 83.23 (70.05–94.63) | <0.001 |
|  | ADC−R(900) | 80.11 (74.91–90.89) | 75.21 (73.14–78.24) | 87.51 (78.92–95.96) | <0.001 |
|  | ADC−R(950) | 86.83 (82.14–93.26) | 82.68 (81.06–84.89) | 90.87 (85.23–97.06) | <0.001 |
|  | ADC−R(1000) | 90.05 (87.51–95.12) | 87.70 (86.68–89.29) | 93.37 (89.52–97.96) | <0.001 |
|  | ADC−R(1050) | 93.23 (91.58–96.72) | 91.71 (91.08–92.71) | 95.45 (92.76–98.61) | <0.001 |
|  | ADC−R(1100) | 95.76 (94.80–98.01) | 94.88 (94.50–95.47) | 97.20 (95.57–99.14) | <0.001 |
|  | ADC−R(1150) | 97.99 (97.54–99.07) | 97.60 (97.40–97.86) | 98.69 (97.94–99.59) | <0.001 |

Data are presented as median value and interquartile range.

^a^, The definition of ADC−R(*x*) is the ratio of voxels with ADC values ranging from 200 × 10^−6^ mm^2^/s to the threshold (*x*).

^b^, P values are based on Mann-Whitney U test for continuous variables.

**Abbreviations**: ADC, apparent diffusion coefficient; IQR, interquartile range
